# Myeloma‐associated hemophagocytic lymphohistiocytosis – A comprehensive case study and a novel chemotherapy‐free approach with anakinra

**DOI:** 10.1002/jha2.975

**Published:** 2024-07-04

**Authors:** Maged Al‐Ammari, Danny Hsu, Adam Bryant

**Affiliations:** ^1^ Department of Haematology Liverpool Hospital Liverpool Australia; ^2^ School of Clinical Medicine University of New South Wales Sydney Australia

**Keywords:** chemotherapy, immunotherapy, macrophages, malignancy, thrombocytopenia

## Abstract

Hemophagocytic lymphohistiocytosis (HLH) is an immune response syndrome characterized by excessive inflammation and tissue destruction. A limited number of cases involving HLH patients with concomitant multiple myeloma (MM), leading to significant mortality, have been documented, underscoring the importance of timely diagnosis. We present the case of a 78‐year‐old previously healthy male admitted to our hospital with a newly diagnosed MM. Subsequently, he was diagnosed with HLH and received treatment with anakinra, intravenous immunoglobulin, and dexamethasone. This case report highlights the unique aspect of being the first documented instance of myeloma‐associated HLH treated with anakinra.

## 1 INTRODUCTION

Hemophagocytic lymphohistiocytosis (HLH) is an immune response syndrome marked by aberrant hyperinflammation, hyperferritinemia, and a potentially fatal cytokine storm, primarily driven by T cells, leading to excessive inflammation and tissue destruction [1].

HLH can be classified into two categories: primary, which is hereditary and may manifest sporadically even without a family history, and secondary, frequently associated with conditions such as infection, malignancy, or autoimmune diseases [2]. The diagnosis of HLH can be confirmed either through a molecular diagnosis consistent with primary HLH or by meeting five out of nine diagnostic criteria used in the HLH‐2004 trial [3, 4]. These criteria encompass fever, splenomegaly, cytopenias affecting two or more of the three blood lineages (hemoglobin [Hb] less than 90 g/L, platelets less than 100 × 10^9^/L, neutrophils less than 1.0 × 10^9^/L), hypertriglyceridemia (fasting triglycerides exceeding 3.0 mmol/L or 265 mg/dL) and/or hypofibrinogenemia (fibrinogen below 1.5 g/L), evidence of hemophagocytosis in bone marrow or spleen or lymph nodes without malignancy, low or absent natural killer (NK) cell activity (according to local laboratory reference), and elevated levels of ferritin (above 500 mg/L) and sCD25 (soluble interleukin‐2 [IL‐2] receptor, above 2400 U/mL). The specific thresholds for abnormal values are integral to the diagnostic process [5].

The original HLH‐94 treatment protocol, although originally conceived for pediatric patients, continues to serve as the established standard of care for adults with the potential for individualized modifications concerning the duration and dosage of the HLH‐94 treatment regimen in the adult population. The HLH‐2004 protocol subsequently introduced early initiation of ciclosporin but did not show any additional benefits in mortality, and hence HLH‐94 remains the standard of care in adults [4, 5]. For adults with primary HLH, consideration for allogeneic hematopoietic stem cell transplantation (alloHSCT) is essential, given its substantial enhancement of outcomes, a benefit that has been particularly pronounced in pediatric cases [6].

Multiple myeloma (MM) is a hematologic malignancy characterized by the malignant proliferation of monoclonal plasma cells within the bone marrow [7]. Accounting for 1% of all cancers and approximately 10% of hematologic malignancies, MM represents a significant medical concern [8].

In patients with malignancy‐induced HLH, the majority of cases (80%) were associated with lymphoma, whereas only 0.24% of cases were linked to MM [9]. Notably, among HLH patients with concomitant MM, there is a significant increase in mortality compared to those without MM [10].

A limited number of cases have been reported, underscoring the association of HLH induced by MM [11, 12, 13, 14, 15, 16, 17, 18, 19, 20].

The HLH‐94 protocol is employed as the primary therapeutic approach for individuals with HLH, demonstrating a 5‐year survival probability of approximately 54% [21]. Anakinra, an IL‐1 receptor antagonist, was initially found to be effective in pediatric patients with secondary HLH in the frontline setting [22, 23]. The use of anakinra in adults with secondary HLH has also been explored, with one study demonstrating a hospital survival rate of 50% when administered in combination with intravenous immunoglobulin and steroids [24], while another reported a hospital survival rate of 69% [25].

## 2 CASE PRESENTATION

We present the case of a 78‐year‐old previously healthy male who sought evaluation at the hematology clinic due to a one‐month history of easy fatigability, significant weight loss, and anorexia. Initial clinical assessments revealed a myriad of abnormalities, including hypercalcemia, acute kidney injury, hyperferritinemia, anemia, thrombocytopenia, and monoclonal gammopathy.

### 2.1 Clinical findings

Upon laboratory evaluation, the patient exhibited the following results: Hb of 104 g/L (130–170), mean corpuscular volume of 83 fl (80–100), white blood count (WBC) of 5.9 × 10^9^/L (4–10), neutrophils at 4.4 × 10^9^/L (2–7), platelets at 81 × 10^9^/L (150–400), corrected calcium at 4.20 mmol/L (2.1–2.6), creatinine at 178 µmol/L (60–110), lactate dehydrogenase at 267 U/L (120–250), Beta2 microglobulin at 10.15 mg/L (0.9–2), triglycerides at 1.4 mmol/L ( = < 2), prothrombin time at 15.0 s (9–13), activated partial thromboplastin time at 32 s (25‐37), and fibrinogen at 1.1 g/L (2–4.6). Hepatic profile indicators included alanine aminotransferase at 22 U/L (10–50), aspartate aminotransferase at 22 U/L (10–35), and albumin at 19 g/L (30–44).

Immunofixation analysis disclosed 12.3 g/L of IgA kappa paraprotein, with normal immunoglobulin levels. Free kappa measured at 1680.00 mg/L, free lambda at 9.99 mg/L, resulting in a kappa/lambda ratio of 168.17. A skeletal survey revealed multiple lytic lesions within the spine, ribs, pelvis, and lower limb extremities.

### 2.2 Bone marrow examination

**FIGURE 1:**
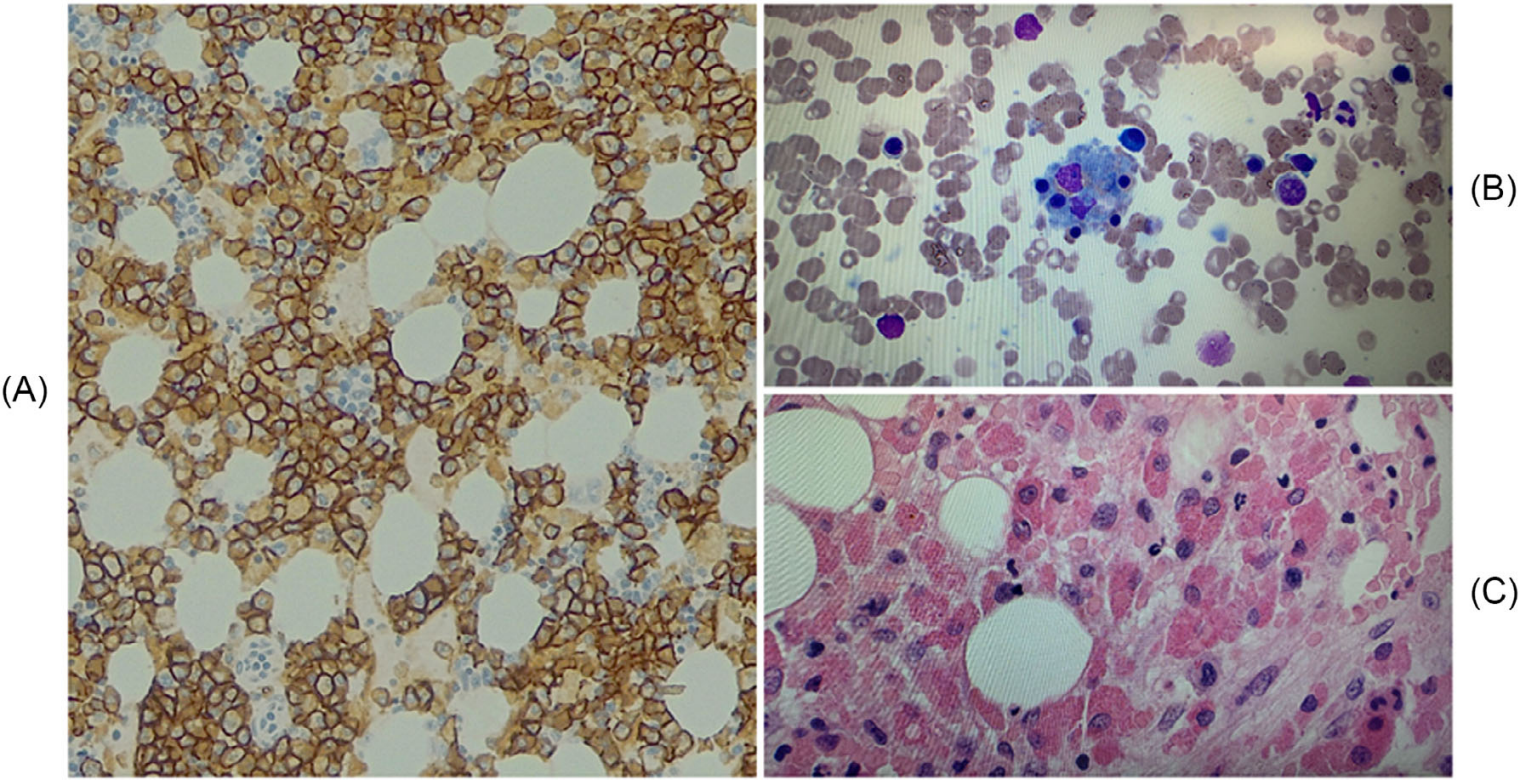
(A) Initial bone marrow trephine shows diffuse infiltrate of CD138 positive plasma cells accounting for 85% of total cellularity. These plasma cells are kappa‐restricted. (B) Follow‐up bone marrow aspirate shows marked haemophagocytic activity with inclusions suggestive of predominant platelet inclusions. (C) Follow up bone marrow trephine, normocellular with an increase in histiocytes and haemophagocytic activity and < 5% plasma cells on a background of known plasma cell myeloma.

Bone marrow aspirates depicted a markedly hypercellular aspirate with 75% plasma cells exhibiting markedly dysplastic forms. The biopsy revealed a diffuse infiltrate of CD138‐positive kappa‐restricted plasma cells, constituting 85% of total cellularity (Figure 1A). SNP chromosome microarray analysis from the bone marrow exhibited cytogenetic abnormalities consistent with plasma cell myeloma, including 1q21 gain, monosomy 13, trisomy 3, 6q deletion, 4p deletion, and 1p deletion. The previous features were consistent with MM R‐ISS stage III.

### 2.3 Treatment and subsequent complications

The patient was admitted, and his hypercalcemia responded well to vigorous intravenous fluid administration and pamidronate. Treatment for concomitant pneumonia, manifested by fever, shortness of breath, cough, and lung consolidation apparent on chest X‐ray, included intravenous antibiotics. Multiple myeloma therapy comprising cyclophosphamide, bortezomib, and dexamethasone was initiated.

However, thrombocytopenia and anemia persisted (despite the cyclophosphamide already having been suspended), and his functional state remained very poor (after around 16 days in hospital), despite a good response in creatinine from 178 to 91 µmol/L, corrected calcium from 3.78 to 1.61 mmol/L, and total protein from 108 to 58 g/L. Furthermore, ferritin levels continued to rise, reaching 35,475 µg/L. Other unusual features indicating a process distinct from active myeloma were observed, as the patient presented signs of volume overload such as bilateral pitting edema, and ascites. Abdominal ultrasound highlighted a normal spleen, small ascites, and heterogeneous echotexture with increased echogenicity of the liver. A liver fibro scan was done and it did not show features of liver fibrosis. Transthoracic echocardiography demonstrated a mildly dilated left ventricle with severe systolic dysfunction, a normal right ventricular size with mild dysfunction, moderate biatrial dilatation, and mild valvular regurgitations, which was surprising given the patient's lack of any prior cardiac history.

Due to suspicion of HLH, the patient underwent further investigation (Table 1). A decision was made to repeat the bone marrow aspirate and biopsy, with the second study revealing prominent histiocytes and hemophagocytic activity (Figure 1B,C). Reduced NK cell function and elevated Epstein‐Barr virus DNA polymerase chain reaction (1030 copies/mL) and Soluble CD25 (1583 pg/ml) further supported the diagnosis of HLH and fulfilled the published diagnostic criteria used in the HLH‐2004 trial [26].

**TABLE 1:** Clinical, laboratory, and radiological features supporting hemophagocytic lymphohistiocytosis (HLH) diagnosis according to the HLH‐2004 trial.

Criteria	Patient values	HLH‐2004 reference values
Temperature	39.3°C	≥38.5°C
Spleen size	10.4 cm (Normal spleen)	Splenomegaly
Hemoglobin	71 g/L	<90 g/L
Platelets	6000/microL	<100,000/microL
Neutrophils	1000/microL	<1000/microL
Fibrinogen	100 mg/dL	<150 mg/dL
Fasting triglycerides	25.2 mg/dL	>265 mg/dL
Hemophagocytosis	Yes (bone marrow)	Hemophagocytosis in bone marrow, spleen, lymph node, or liver
NK cell activity	Reduced compared to the control	Low or absent NK cell activity
Ferritin	35,475 ng/mL	>500 ng/mL
sCD25 (soluble IL‐2 receptor)	1583pg/ml	>2 SD from the mean, mean = 533pg/ml (242‐1043)

Abbreviations: NK, natural killer; sCD25, soluble CD25; SD, standard deviation.

### 2.4 Treatment for HLH and subsequent clinical course

It was notable that the progressive HLH occurred despite the patient having had (and responded to) effective therapy for their myeloma, including dexamethasone 20 mg weekly. In response to the HLH diagnosis, the patient was initiated on dexamethasone 10 mg/m^2^ daily, anakinra 100 mg subcutaneously daily, and IVIG 1gm/kg for two days. In the follow‐up clinic visit approximately 3 weeks later, the patient, who had completed a 2‐week course of anakinra and was on a tapering dose of dexamethasone, exhibited improvement in overall condition. Notably, platelet count (PLT) increased to 100 × 10^9^/L, Hb rose to 128 g/L, Ferritin levels decreased to 2790 µg/L, and anakinra was ceased. Approximately six months later, the patient is currently ambulatory and being followed as an outpatient. He is experiencing a very good partial response, with a normal free light chain ratio and no detectable paraprotein. His creatinine is 93 µmol/L, Hb is 96 g/L, and PLT is 124 × 10^9/L.

## 3 DISCUSSION

In this report, we present an atypical clinical case involving MM concomitant with HLH, alongside manifestations of heart failure and suspected liver fibrosis.

Myeloma‐associated HLH is a rare occurrence [9], with only 10 documented cases reported previously. Lenalidomide‐related HLH has been reported in patients with MM, with successful outcomes following the cessation of lenalidomide and appropriate HLH therapy [17, 20]. There has been no data current about HLH correlated with cyclophosphamide or bortezomib. The case presented herein constitutes the eleventh documented case. Notably, this case stands out as the first among the reported cases to be treated with anakinra, marking a unique therapeutic approach within the existing literature.

Diagnosing myeloma‐associated HLH is intricate, as the diagnostic criteria often overlap with manifestations of malignancy or infections. In our case, the initial bone marrow analysis did not reveal evidence of hemophagocytosis; instead, it demonstrated a diffuse infiltrate composed of clonal plasma cells, constituting 85% of the total cellularity. However, the subsequent bone marrow evaluation confirmed the presence of hemophagocytosis. Consistent with previously documented cases [11, 14, 16, 18], our observation reveals a pattern where the initial bone marrow assessment lacks evidence of hemophagocytosis, which becomes evident upon subsequent evaluation. Two of the previously reported cases showed only clonal plasma cells with the initial bone marrow evaluation and hemophagocytosis features were apparent with the subsequent bone marrow evaluation as the clonal plasma cells improved [14, 18]. Consistent with our reported case, the emergence of histiocytes and hemophagocytic activity in the subsequent bone marrow evaluation coincided with a reduction of clonal plasma cells to less than 5%. This underscores the diagnostic challenges, in that hemophagocytic activity may have been obscured by the presence of clonal plasma cells during the initial bone marrow evaluation. Alternatively, the HLH may have developed subsequent to the commencement of therapy for the patient's MM. While ultimately this is extremely difficult to distinguish, a careful retrospective review of the initial diagnostic marrow did not identify morphological features of HLH. Although treatment of the underlying cause is typically of prime importance in managing secondary HLH, the patient had already received two doses of bortezomib (2 weeks of therapy), and the issue with the overt cytopenias appeared progressive. Therefore, the treating team believed that another modality was required to attain control of the HLH.

Four previously reported patients manifested HLH subsequent to autologous HSCT for MM [12, 15, 17, 20]. The onset of HLH occurred approximately three months post‐transplantation in three patients. Notably, two patients exhibited concurrent MM progression, while the others did not (Table 2). This observation suggests a potential immunological association between HLH and MM, rather than HLH merely presenting as a consequence of MM. This noteworthy finding underscores the relevance of immunotherapeutic approaches, such as anakinra, as exemplified in our reported case.

**TABLE 2:** Clinical characteristics of published case reports of myeloma‐associated hemophagocytic lymphohistiocytosis (HLH) post autologous stem cell transplant.

Sex/ Age (years)	MM stage**^a^**	HLH presentation	Concurrent MM progression	Possible HLH triggers	HLH therapy	HLH outcome	References
F/54	Stage IIIA	16 days after ASCT	No	Incomplete immune reconstitution after ASCT	IVIG, Dexamethasone	BM 39 days after ASCT without evidence of haemophagocytosis	Ostronoff et al. [15]
M/59	Stage IIIA	126 days after ASCT	Yes	Incomplete immune reconstitution after ASCT, local progression of MM, anticonvulsants	IVIG, cortisol, etoposide	Aggressive, non‐responsive to HLH therapy	Machaczka et al. [12]
F/70	Not given	Three years after ASCT	No	Lenalidomide related	Withhold lenalidomide, Dexamethasone	At 8 weeks, normalization of blood counts	Runge et al. [17]
Not given/29	Not given	Three months after ASCT	Yes	Lenalidomide related, MM progression	Withhold lenalidomide, HLH‐2004 protocol	HLH resolved with an incomplete recovery of hematopoiesis, dependent on platelet transfusion	Milczarek et al. [20]

^a^ MM stage at presentation, according to Salmon–Durie classification.

Abbreviations: ASCT, autologous stem cell transplant; BM, bone marrow; F, female; HLH, hemophagocytic lymphohistiocytosis; IVIG, intravenous immunoglobulin; M, male; MM, multiple myeloma.

Among the previously documented cases, a spectrum of organ involvement was observed. Three cases exhibited acute liver failure [16, 18, 19], concomitant with acute renal failure seen in an equivalent number of cases [12, 18, 19]. Acute respiratory failure manifested in one case [12], while disseminated intravascular coagulation was reported in one case [18]. Additionally, subarachnoid hemorrhage occurred in a distinct case [12]. These varied organ‐specific complications align with the findings of liver and heart dysfunction noted in our case. This diverse organ involvement emphasizes the intricate and multi‐system nature of complications associated with myeloma‐associated HLH, underscoring the need for comprehensive and tailored management strategies.

In a comprehensive review of treatment modalities for myeloma‐associated HLH, various therapeutic approaches were employed. Pomalidomide and daratumumab demonstrated efficacy in a patient who had previously failed etoposide‐based treatment [11]. A patient exhibited resistance to the combination of etoposide, steroid, and intravenous immunoglobulin (IVIG) [12]. Conversely, successful outcomes were observed with distinct therapeutic regimens, including isatuximab, bendamustine, and dexamethasone [20], vinblastin, adriamycin, and dexamethasone [13], and etoposide, steroid, and cyclosporin A [14]. IVIG therapy also contributed to improvement in another case [15]. Tragically, a patient deteriorated following etoposide treatment and eventually succumbed [16]. Among cases treated with steroid therapy, outcomes varied, with one patient demonstrating improvement while the other two experienced deterioration [17, 18, 19]. Significantly, patients treated with immunotherapeutic agents showed favorable responses, mirroring the positive outcome observed in our reported case with IVIG, steroid, and anakinra treatment.

It is noteworthy that although the patient received a chemotherapy regimen incorporating steroids, it proved inadequate in managing the HLH. This underscores the imperative to entertain alternative diagnoses in cases where patients do not exhibit improvement while undergoing efficacious MM therapy. This highlights the potential efficacy of immunotherapeutic agents in MM‐associated HLH, in the absence of a standard treatment for these patients, necessitating further investigation.

Anakinra, an IL‐1 antagonist has been increasingly used in the setting of secondary HLH in adults given its safety profile and reported efficacy in limited studies [27, 28]. During the treatment of adult secondary HLH, anakinra is generally well tolerated. However, grade three liver injury occurred in 6% of patients, which improved upon discontinuation of the drug [27]. Other reported side effects included injection‐site pain, erysipelas, pneumonia, and allergic reactions [28]. In addition, anakinra has been used to manage critically ill patients with suspected reactive HLH in the intensive care unit. When used with IVIG and steroids, it has shown positive outcomes, with a 50%–69% hospital survival rate [24, 25]. The patient's assessment indicated a positive response to MM treatment, as evidenced by repeated bone marrow assessments and biochemical features. Consequently, we decided to avoid using etoposide at this stage, to avoid its myelosuppressive effects, and preserve it as a potential salvage therapy for the HLH in case of him not responding to the chemotherapy‐free approach. Our patient appeared to have responded favorably to this immunomodulatory approach without the need for etoposide‐based treatments which raises the provocative question of the role of a chemotherapy‐free approach to myeloma‐associated HLH.

In conclusion, our reported case highlights the rarity and challenging diagnosis of myeloma‐associated HLH and presents a unique case treated with anakinra, offering a novel therapeutic avenue.

## AUTHOR CONTRIBUTIONS

Maged Al‐Ammari compiled the case report, conducted the literature, and wrote the manuscript. Danny Hsu and Adam Bryant identified the novelty of the case and wrote the manuscript.

## CONFLICT OF INTEREST STATEMENT

The authors declare no conflict of interest.

## FUNDING INFORMATION

The authors received no specific funding for this work.

## ETHICS STATEMENT

The authors have confirmed ethical approval statement is not needed for this submission.

## PATIENT CONSENT STATEMENT

The patient has signed written informed consent.

## CLINICAL TRIAL REGISTRATION

The authors have confirmed clinical trial registration is not needed for this submission.

## Data Availability

The data that support the findings of this study are available from the corresponding author upon reasonable request.
